# Effects of the Brazilian Native Fruit Jaboticaba (*Plinia cauliflora*) Peel on Inflammatory and Oxidative Stress Pathways: Insights from a Pilot Study in Hemodialysis Patients and Renal Cell Models

**DOI:** 10.3390/foods14234030

**Published:** 2025-11-24

**Authors:** Ligia Soares Lima, Jessyca Sousa de Brito, Marcelo Ribeiro-Alves, Karen Salve Coutinho-Wolino, Rodrigo dos Santos P. Duarte, Rafael Hospodar Felippe Valverde, Marcelo Einicker-Lamas, Andresa A. Berretta, Carmen Lucía Sanz, Lia S. Nakao, Peter Stenvinkel, Denise Mafra

**Affiliations:** 1 Carlos Chagas Filho Institute of Biophysics, Federal University of Rio de Janeiro (UFRJ), Rio de Janeiro 21941-902, RJ, Brazil; ligialima@biof.ufrj.br (L.S.L.); jessyca.sousa.brito@gmail.com (J.S.d.B.); valverde@biof.ufrj.br (R.H.F.V.); einicker@biof.ufrj.br (M.E.-L.); 2 HIV/AIDS Clinical Research Center, National Institute of Infectious Diseases Evandro Chagas (INI/Fiocruz), Rio de Janeiro 21040-360, RJ, Brazil; 3 Chair Propolis Working Group, International Honey Commission (IHC), Ribeirão Preto 14020-670, SP, Brazil; 4 Department of Basic Pathology, Federal University of Paraná, Curitiba 81531-980, PR, Brazil; 5 Division of Renal Medicine, Department of Clinical Science, Technology and Intervention, Karolinska Institutet, 141 86 Stockholm, Sweden; 6 Graduate Program in Nutrition Sciences, Fluminense Federal University (UFF), Niterói 24070-090, RJ, Brazil

**Keywords:** renal dialysis, chronic kidney disease, inflammation, oxidative stress, jaboticaba, *Plinia cauliflora*

## Abstract

Jaboticaba (*Plinia cauliflora*), a Brazilian native fruit rich in bioactive compounds, exhibits potent anti-inflammatory and antioxidant properties. This pilot study evaluated the effects of jaboticaba peel supplementation on inflammatory and oxidative stress markers and uremic toxins among patients with chronic kidney disease (CKD) undergoing hemodialysis (HD) and explored its molecular effects in LLC-PK1 renal cells. A randomized, controlled clinical trial was conducted with 27 patients (55.0 [19.5] years, BMI 24.3 [3.8] kg/m^2^) on regular HD. Participants were allocated to receive the jaboticaba peel formulation (3.3 g/day, equivalent to ~667 mg of phenolic compounds) for 3 weeks or to routine treatment (control). Plasma levels of interleukin (IL)-1β and IL-17E (ELISA), lipid peroxidation (TBARS), protein carbonylation, and plasma levels of uremic were analyzed. LLC-PK1 cells were treated with 100 µL of jaboticaba peel formulation at different concentrations, and a panel of inflammatory genes was evaluated. While plasma IL-1β and IL-17E concentrations were increased in the control group, the jaboticaba group exhibited no significant changes, suggesting anti-inflammatory protection. Transcriptomic analysis revealed downregulation of key components of the TLR–MYD88–NF-κB–IL-1 axis after cell treatment. Additionally, cells treated with jaboticaba formulation (1.5%) showed reduced ROS levels, indicating antioxidant capacity. In conclusion, supplementation with jaboticaba peel attenuated the increase in pro-inflammatory markers in HD patients. These results suggest that jaboticaba peel holds promise as an adjuvant nutritional intervention for chronic inflammation in CKD.

## 1. Introduction

Plant-derived bioactive compounds have gained increasing attention for their anti-inflammatory and antioxidant properties, aligning closely with the modern “food as medicine” paradigm [1,2]. Jaboticaba (*Plinia cauliflora*), a Brazilian native fruit, presents a high concentration of phenolic compounds, particularly anthocyanins, ellagic acid, and hydrolyzable tannins, mainly concentrated in its peel and seeds, which are considered the primary contributors to its anti-inflammatory and antioxidant effects [3,4,5,6,7]. The peel contains a complex phytochemical profile, primarily composed of cyanidin-3-glucoside, dephinidin-3-glucoside, ellagitannins (such as vescalagin and castalagin), and gallic acid derivatives, which contribute to its high antioxidant capacity and anti-inflammatory activity. These bioactive compound constituents are primarily responsible for the fruit’s health benefits, and their concentration in the peel is markedly higher than in the pulp, supporting the rationale for selecting the peel as the source material in the present study [4,6].

Previous studies have shown that in rats fed a high-fat diet, supplementation with jaboticaba extract attenuated the expression of key inflammatory mediators, including Toll-like receptor 4 (TLR-4), tumor necrosis factor-alpha (TNF-α), and nuclear factor-κB (NF-κB) in the colon [8]. Additionally, administering jaboticaba peel and seed powder (5%, 10%, and 15% *w*/*w*) for four weeks reduced circulating levels of interleukin (IL)-6 and TNF-α in obese mice [9]. Consistent with these findings, dietary supplementation with freeze-dried jaboticaba peel powder in high-fat-fed mice also reduced hepatic levels of IL-1β and IL-6, as well as phosphorylated IκB-α, indicating inhibition of NF-κB pathway activation [10].

Since systemic inflammation plays a pivotal role in the onset and progression of chronic kidney disease (CKD), these findings suggest that jaboticaba may hold therapeutic potential in this context [11]. CKD is defined by progressive loss of kidney function and represents a significant public health challenge. In 2021, CKD affected over 670 million people worldwide, with more than 19 million new cases and over 1 million deaths attributed to the disease [12]. Between 1990 and 2021, CKD rose from the 29th to the 18th leading cause of death globally [13,14,15]. By 2040, CKD is projected to be among the five most common causes of death. Its high prevalence, associated clinical complications, and substantial healthcare costs are closely linked to pathophysiological mechanisms that sustain renal injury, including chronic low-grade inflammation and oxidative stress. These factors contribute to CKD progression and to the increased cardiovascular morbidity and mortality observed in these patients [16,17].

In CKD, inflammation is sustained by multiple interrelated triggers, including oxidative stress, gut dysbiosis, uremia, dyslipidemia, and volume overload [18,19]. In patients undergoing hemodialysis (HD), additional factors, such as blood–membrane interactions, dialysate contamination, and uremic toxin accumulation, further amplify the inflammatory state [20,21]. These stimuli activate innate immune receptors, such as TLRs and NOD-like receptors (NLRs), and downstream signaling pathways, most notably NF-κB, leading to increased production of pro-inflammatory cytokines, such as IL-1β, IL-6, TNF-α, and IL-17. These mediators recruit immune cells and activate renal resident cells, promoting oxidative stress, fibrosis, and progressive loss of kidney function [22,23,24,25,26]. Given its critical role in disease progression and cardiovascular complications, modulation of inflammation and oxidative stress represents a significant therapeutic target [1,2].

Preclinical data suggest that jaboticaba phenolics may modulate inflammatory pathways relevant to CKD complications [8,27,28]. However, based on the available evidence, no clinical trials have examined the effects of jaboticaba peel supplementation in patients with CKD following HD treatment. Therefore, the present pilot study aimed to investigate whether supplementation with jaboticaba peel can influence markers of inflammation and oxidative stress in individuals with CKD undergoing HD. To complement the clinical data, in vitro assays and transcriptomic analyses were conducted to explore potential cellular and molecular mechanisms underlying the observed effects.

## 2. Materials and Methods

### 2.1. Subjects

This pilot study was a randomized, longitudinal, controlled clinical trial with a parallel-group design. The study was conducted from October to December 2024 in patients undergoing regular hemodialysis treatment (3 times/week, 4 h/session) at Prodoctor Sistema Integrado de Saúde (Rio de Janeiro, RJ, Brazil).

The sample size calculation was performed using G*Power 3.1, considering a statistical power of 80%, a two-tailed significance level of 5%, and an effect size of 1.32, based on NF-κB expression as the primary outcome, as reported by Alvarenga et al. (2020) [29]. The final calculated sample consisted of 30 patients, allocated into two groups: the jaboticaba group (*n* = 15) and the Control Group (*n* = 15). The study was conducted in accordance with the Declaration of Helsinki, and the study protocol was approved by the Research Ethics Committee of the Faculty of Medicine, Fluminense Federal University (UFF) (approval number 6.953.399) and registered at ClinicalTrials.gov (NCT06394531). Written informed consent was obtained from all participants before data collection, after which demographic, clinical, and biochemical information were collected.

### 2.2. Inclusion and Non-Inclusion Criteria

Inclusion criteria were patients in stage 5 of CKD (estimated glomerular filtration rate < 15 mL/min), undergoing regular HD for more than six months, aged between 18 and 75 years, of both sexes, using an arteriovenous fistula (AVF) as vascular access, and following an individualized dietary prescription (energy intake of 25–35 kcal/kg/day and protein intake of 1.0–1.2 g/kg/day), according to the National Kidney Foundation (NKF-KDOQI) guidelines [30]. For recruitment, non-inclusion criteria factors were patients with autoimmune and/or infectious diseases, diabetes, cancer, AIDS, pregnancy, use of catabolic drugs or antibiotics, and intake of antioxidant supplements, prebiotics, probiotics, or symbiotics.

### 2.3. Randomization Implementation and Blinding

Participants were randomized in pair blocks according to age, sex, time on dialysis, and body mass index (BMI). The random sequence of supplementation (jaboticaba or control) was generated by an independent researcher using computer-based randomization (1:1 allocation ratio). Laboratory analyses were centralized and performed in a blinded manner.

### 2.4. Composition of Jaboticaba Formulation

The dosage of jaboticaba peel supplementation was based on previous efficacy and safety studies using hydroethanolic extracts of jaboticaba peel in animal and in vitro models, complemented by clinical studies employing jaboticaba-derived products [31,32,33]. Using non-clinical data and applying the U.S. Food and Drug Administration (FDA) guidelines for dose translation from animals to humans, the selected dosage was 3.3 g/day of dried peel extract (equivalent to approximately 667.0 mg/day of phenolic compounds) [34].

Our research collaborator obtained fresh fruits purchased at a local market, developed the extraction process, and formulated it. The jaboticaba peel formulation was prepared and analytically characterized at Apis Flora^®^ (Ribeirão Preto, SP, Brazil), in collaboration with the research team. The formulation was not commercially available and was produced explicitly for research purposes. The hydroalcoholic solution used for the extraction of jaboticaba peel consisted of 96% ethanol (*v*/*v*). A total of 60 kg of fresh jaboticaba peel was used in the process, which involved maceration followed by percolation. The final yield of the jaboticaba peel extract was 125 kg, corresponding to 3.5–4.5% of total dry matter and an ethanol content of 65 °GL.

The crude extracts were filtered through filter paper under reduced pressure and stored in amber flasks at −20 °C. The jaboticaba peel formulation used in the intervention was composed of jaboticaba peel extract, xanthan gum, potassium sorbate, stevia (as a sweetener), ascorbic acid, natural grape flavor, and purified water. The formulation (density of 1.103 and pH 3.12) was characterized and provided 23.13% *w*/*w* of total dry matter, 43.17 mg/g of total phenolics as gallic acid, and 49.94 mg of monomeric anthocyanins as cyanidin/100 g.

#### 2.4.1. Chemical Characterization

##### Determination of Total Phenolic Content

The total phenolic content of the samples was determined using the Folin–Denis spectrophotometric method [35], with gallic acid as the standard. Absorbance readings were performed in a Shimadzu UV–Vis spectrophotometer (Shimadzu Corporation, Kyoto, Japan) at a wavelength of 760 nm. Results were expressed as milligrams of gallic acid equivalents per gram of sample (mg GAE/g).

##### Preparation of the Gallic Acid Calibration Curve

A stock solution of gallic acid (0.4 mg/mL) was prepared in distilled water. Appropriate aliquots of this solution were transferred into 50 mL volumetric flasks containing 30 mL of distilled water to obtain standard solutions with final concentrations ranging from 3.2 to 4.8 µg/mL.

To each flask, 2.5 mL of the Folin-Denis reagent and 5.0 mL of 35% (*w*/*v*) sodium carbonate solution were added, and the volume was made up to 10.0 mL with distilled water. The solutions were mixed and allowed to stand in the dark for 30 min before absorbance was measured against a reagent blank. The calibration curve was constructed by plotting absorbance versus gallic acid concentration.

##### Sample Preparation

An aliquot of 0.7 g of the jaboticaba shot sample was weighed into a 50 mL volumetric flask, and the flask was filled to volume with distilled water. The mixture was homogenized, filtered, and 0.5 mL of the filtrate was treated under the same conditions described for the standard. After 30 min of incubation in the dark, absorbance was measured at 760 nm.

#### 2.4.2. Determination of Monomeric Anthocyanin Content

##### Preparation of Buffers for the Total pH Differential Method

Standardized buffer solutions to pH 4.0 and 7.0 were prepared as follows: pH 1.0 buffer (potassium chloride, 0.025 M), 1.86 g potassium chloride was weighed into a beaker, distilled water was added to ca 980 mL, pH was adjusted to pH 1.0 with HCl before being made up to 1 L; pH 4.5 8 buffer (sodium acetate, 0.4 M), 54.43 g sodium acetate was weighed into a beaker, and distilled water was added ca 960 mL, the pH was adjusted to 4.5 pH with HCl before being made up to 1 L.

##### Spectrometry (pH Differential Method)

The quantification of monomeric anthocyanins was performed according to the pH-differential method described in AOAC Official Method 2005.02 [36,37,38]. Approximately 1.0 g of sample was accurately weighed into a 25 mL volumetric flask, and the volume was made up to the mark with distilled water.

Two sample solutions of the shot were prepared by diluting 2.0 mL of the stock solution to 10 mL with buffer solutions at pH 1.0 (potassium chloride buffer, 0.025 M) and pH 4.5 (sodium acetate buffer, 0.4 M), respectively. The solutions were mixed thoroughly and allowed to stand at room temperature for 15 min.

The absorbance of each solution was measured at 510 nm (maximum absorption) and 700 nm using a Shimadzu UV–Vis spectrophotometer (Shimadzu Corporation, Kyoto, Japan), with distilled water as a blank. The absorbance difference between pH 1.0 and pH 4.5 was calculated using the following equation: A = (A510 − A700) pH 1.0 − (A510 − A700) pH 4.5. The anthocyanin content was expressed as mg cyanidin-3-glucoside equivalents per 100 g of sample (mg C3G/100 g).

### 2.5. Experimental Design

Patients in the jaboticaba group received bottles containing the jaboticaba ethanolic peel formulation, along with a dosing syringe. Supplementation consisted of once-daily ingestion of 14 mL of formulation, diluted in 50 mL of water, as specified by the manufacturer (Apis Flora^®^).

On dialysis days, supplementation was supervised by a member of the research team and administered during the final hour of the session; on non-dialysis days, participants were instructed to consume the supplement 1 h after lunch. An instruction leaflet with details on dilution and intake was provided to participants at the beginning of each supplementation period. Throughout the study, the research team maintained contact during site visits and remote check-ins to reinforce adherence and monitor for adverse effects or intolerance. The intervention lasted 3 weeks, and blood samples were collected at baseline and at the end of each phase.

### 2.6. Primary and Secondary Outcomes

The primary outcome was the effect of jaboticaba peel supplementation on plasma concentrations of interleukin (IL)-1β, IL-17E, malondialdehyde (MDA), and protein carbonyls in patients with CKD undergoing HD. Secondary outcomes included effects on biochemical parameters.

### 2.7. Anthropometric Measurement

BMI was calculated using body weight measured after the HD session (dry weight) divided by the square of the individual’s height. Classification followed the World Health Organization (WHO) criteria [39]. All anthropometric measurements were performed by a single registered dietitian trained and experienced in the clinical nutritional assessment of hemodialysis patients. Standardized procedures and calibrated equipment were used throughout the study to ensure measurement accuracy and consistency.

### 2.8. Blood Collection and Biochemical Analyses

Blood samples were collected in the morning, before the HD procedure, and immediately after AVF puncture. Samples were drawn into Vacutainer tubes containing ethylenediaminetetraacetic acid (EDTA; 1.0 mg/mL) and into Vacutainer serum separator tubes (SST II Advance, BD, Sumter, SC, USA). Samples were stored at 2–6 °C and transported to the Clinical Research Unit (UPC–UFF), where they were centrifuged at 3500 rpm for 15 min at 4 °C to separate serum and plasma, which were then stored at −80 °C until analysis. Biochemical analyses, including albumin, phosphorus, potassium, magnesium, and C-reactive protein (CRP), as well as glucose, were performed using an automatic clinical chemistry analyzer (BioClin, Quibasa-Bioclin, Belo Horizonte, MG, Brazil) and corresponding BioClin reagent kits, following the manufacturer’s instructions. All serum biomarkers were measured using enzymatic colorimetric or turbidimetric methods, depending on the analyte.

### 2.9. Assessment of Oxidative Stress Biomarkers

Oxidative stress was evaluated by measuring lipid peroxidation and protein carbonylation. Lipid peroxidation was assessed by determining thiobarbituric acid-reactive substances (TBARS) using an adaptation of the method described by Ohkawa [40]. In brief, 70 μL of plasma or diluted MDA standards were added with 35 μL of 8.1% (*w*/*v*) SDS, 385 μL of 1% (*v*/*v*) phosphoric acid, and 210 μL of 0.6% (*w*/*v*) thiobarbituric acid. After vigorous mixing, the samples were heated in a dry water bath at 95 °C for 1 h, then centrifuged at 4000 rpm for 20 min at 20 °C. An aliquot of 200 μL of the supernatant was transferred in duplicate to a 96-well microplate, and the absorbance was read at 532 nm using a Multiskan GO microplate spectrophotometer (Thermo Fisher^®^, Waltham, MA, USA). MDA levels were expressed as nanomoles per milliliter.

Protein carbonyl content was assessed using an adaptation of the method of Levine et al. (1990) [41]. Briefly, 50 μL of plasma were precipitated with 10% trichloroacetic acid (TCA), and centrifuged at 2000× *g* for 2 min. The pellet was incubated for 15 min at room temperature with 500 μL of either 2 M HCl (blank) or 0.2% (*w*/*v*) 2,4-dinitrophenylhydrazine (DNPH) prepared in 2 M HCl. Samples were vortexed halfway through (7 min) and at the end of incubation. Proteins were reprecipitated with 10% TCA, centrifuged, and washed twice with 1 mL of an ethanol-ethyl acetate (1:1, *v*/*v*) mixture. The final pellet was solubilized in 400 μL of 6 M guanidine hydrochloride, pH 2.3, and centrifuged at 5000× *g* for 3 min. The absorbance of the supernatant was read at 370 nm using a Multiskan GO microplate spectrophotometer (Thermo Fisher^®^, Waltham, MA, USA). Protein carbonyl content was expressed as μmol carbonyl per mg of protein, and the Bradford assay determined total protein concentration.

### 2.10. Assessment of Inflammatory Cytokines

Plasma IL-1β and IL-17E concentrations were determined by quantitative sandwich enzyme-linked immunosorbent assay (ELISA) using commercial development kits (Human IL-1β Standard TMB ELISA Development Kit, Cat# 900-T95K, and Human IL-17E Standard TMB ELISA Development Kit, Cat# 900-T234K; PeproTech Inc., Rocky Hill, NJ, USA), according to the manufacturer’s instructions. All capture, detection, and standard antibodies used in the assays were purchased from PeproTech as components of these commercial kits. Detection ranges were 8–1000 pg/mL for IL-1β and 16–4000 pg/mL for IL-17E.

Briefly, capture antibodies were diluted in phosphate-buffered saline (PBS, pH 7.2) to final concentrations of 0.125 μg/mL for IL-1β and 0.25 μg/mL for IL-17E. After, the captured antibodies were added to coat MaxiSorp 96-well flat-bottom microplates (Nunc, Fisher Scientific, Leicestershire, UK) overnight at room temperature. After overnight incubation, the plates were aspirated and washed, and then the block buffer was added for 1 h and 30 min at room temperature. After blocking, the plates were rewashed, and the serially diluted standards and samples were added for a 2 h incubation at room temperature. Subsequently, the plate was aspirated, rewashed, and a detection antibody was added at 0.25 μg/mL for IL-1β and IL-17E, and incubated for 2 h at room temperature. After additional washing, streptavidin conjugate was added at final concentrations of 0.10 μg/mL for IL-1β and 0.05 μg/mL for IL-17E, and the mixture was incubated for 30 min at room temperature. Tetramethylbenzidine (TMB) substrate was added and incubated for 20 min at room temperature to facilitate color development. The reaction was quenched with 1 M HCl. Absorbance was measured at 450 nm using a Multiskan GO microplate spectrophotometer (Thermo Fisher, Waltham, MA, USA). The results were log-transformed.

### 2.11. Uremic Toxins Analysis

Evaluation of gut microbiota-produced uremic toxins, including indole-3-acetic acid (IAA), p-cresyl sulfate (p-CS), and indoxyl sulfate (IS), was performed using high-performance liquid chromatography (HPLC) coupled with fluorescence detection. Serum samples, after preparation, were analyzed in a Shimadzu HPLC platform equipped with a quaternary pump, autosampler, and fluorescence detector operated via LC Solution software (version 1.25 SP5). Sample processing followed the protocol described by Meert et al. (2012) [42], with minor adaptations. Briefly, 100 µL of plasma was diluted, heated, rapidly cooled on ice, and centrifuged at 1300× *g* for 20 min at 4 °C. The supernatant obtained was subsequently ultrafiltered through a 30 kDa cutoff membrane, and a 10 µL aliquot of the ultrafiltrate was injected into the chromatographic system. Separation was achieved using a Shimadzu Prominence system fitted with a C8 Luna column and a gradient of ammonium formate and methanol. Fluorescence detection was carried out at specific excitation/emission wavelengths optimized for each analyte [43].

### 2.12. Cell Culture

To evaluate the effects of jaboticaba peel formulation on renal cells, a porcine kidney proximal tubule cell line (LLC-PK1) was purchased from American Type Culture Collection (ATCC). The LLC-PK1 cells were grown in Dulbecco Modified Eagle Medium (DMEM, low glucose) supplemented with 10% inactivated fetal bovine serum (FBS) and 1% antibiotic solution (penicillin and streptomycin at 100 IU/mL and 100 µg/mL, respectively). Cell cultures were kept at 37 °C under a humidified atmosphere containing 5% CO_2_.

### 2.13. MTT Cellular Viability Assay

The assay was performed according to the manufacturer’s instructions. Briefly, cells (2 × 10^4^) were seeded into 96-well plates (100 µL of cell suspension per well). After 24 h, 100 µL of each jaboticaba peel formulation solution, prepared at concentrations ranging from 0.022 to 0.0006875 mg/mL, was added to the respective wells. The control solutions included a 60% (*v*/*v*) ethanol solution (matching the formulation hydroalcoholic content) and a negative control (untreated cells) [44].

After 24 h of incubation at 37 °C in a humidified atmosphere with 5% CO_2_ and protected from light, the test solutions were replaced with 180 µL of low-glucose DMEM containing 20 µL of MTT solution (5 mg/mL). The plates were incubated under the same conditions for 3 h, then centrifuged at 1218× *g* for 7 min. Formazan crystals were dissolved in 200 µL of dimethyl sulfoxide (DMSO), and absorbance was measured at 490 nm in a multiwell plate reader (TP-Reader, Thermo Plate^®^, Shenzhen, China).

Cell viability was calculated relative to untreated and ethanol-treated control cells, based on mean values from four independent experiments performed in quintuplicate. The percentage of viable cells was determined using GraphPad Prism 5 Software.

### 2.14. Determination of Reactive Oxygen Species (ROS) Levels

Cells were seeded into 96-well plates at a density of 2 × 10^4^ cells per well. After 24 h of incubation at 37 °C in a humidified atmosphere containing 5% CO_2_, jaboticaba peel formulation was added at a concentration of 0.0006875 mg/mL. The following control groups were included: a negative control (DMEM with 10% fetal bovine serum, FBS), a solvent control (1.5% hydroethanolic solution at 60% *v*/*v*), an antioxidant control (Butylated Hydroxyanisole, BHA), and a positive control (600 µM hydrogen peroxide incubated for 30 min).

After the designated incubation period, cell supernatants were removed, and 30 µM of 2′,7′-Dichlorofluorescein diacetate (H_2_DCFDA) was added to each well. Following a 30-min incubation, wells were washed twice with 100 µL of PBS. Finally, 100 µL of PBS was added to each well, and fluorescence was measured using a spectrofluorometer (SpectraMax M5) at excitation/emission wavelengths of 495/530 nm, respectively, as previously described [45].

### 2.15. Transcriptomic Data Reanalysis of Public RNA-Seq Datasets

Publicly available RNA-seq datasets (accession numbers GSE171311 and GSE171618; *Homo sapiens*, expression profiling by high-throughput sequencing) were sourced from the Gene Expression Omnibus (GEO) database. Raw and processed data were analyzed in R (version 4.5.1) using RStudio (version 2025.09.1-401; Posit, PBC). Raw count matrices were imported and processed with DESeq2 (v1.42.0) for differential gene expression analysis. For the heatmap representation, raw expression values were log_2_-transformed, and normalized data were used for visualization.

A pre-specified panel of inflammatory genes was defined a priori, comprising the following targets: TLR1, TLR2, TLR3, TLR4, TLR5, TLR6, NOD1, AIM2, MEFV, RIPK2, IRAK1, MYD88, TRAF6, TYK2, JAK1, JAK2, STAT3, MAP3K7 (TAK1), TAB1, CHUK (IKKα), IKBKB (IKKβ), NFKBIA, NFKBIB, NFKBIE, NFKB1, NFKB2, REL, RELA, RELB, IL1A, IL1B, IL6, IL8, IL12A, IL12B, IL17A, IL18, TNF, and CCL2. This list was designed to capture canonical innate immune pathways implicated in CKD-related inflammation.

Differential expression between ellagic acid-treated and control samples was assessed using Wald tests on estimated parameters from a negative binomial generalized linear model implemented in the DESeq2 package in R Studio (v. 2025.05.0). Genes with a false discovery rate (FDR), adjusted *p* < 0.05, and an absolute log_2_ fold change ≥+1.0 or ≤−1.0 were considered significantly regulated.

Normalized expression values for the inflammatory gene panel were converted to z-scores per gene across samples and visualized as heatmaps using *pheatmap* (v.1.0.12), with hierarchical clustering applied to rows to group genes with similar expression profiles.

Volcano plots were generated by plotting log_2_ fold change (Ellagic Acid vs. Control) against −log_10_ (FDR), with red, blue, and grey denoting upregulated, downregulated, and non-significant genes, respectively. For each gene, log_2_ fold change, nominal *p*-value, FDR, and regulation category were summarized in the accompanying table.

### 2.16. Statistical Analysis

The assumption of normality was verified by analyzing model residuals using graphical methods (Q–Q plots and histograms) and descriptive statistics rather than formal hypothesis testing, given the small sample size. Categorical variables were summarized as percentages, and continuous variables as medians with interquartile ranges (IQRs). Between-group comparisons were performed using the Mann–Whitney U test for continuous variables and the Chi-square test for categorical variables. To assess time-by-intervention effects, linear mixed-effects models were employed, treating participants as random effects. Fixed effects included age, sex, time on dialysis, and BMI. For comparisons between the baseline and post-intervention periods, multiple linear mixed-effects models were used, with patient identification serving as a random effect to account for replicated measures. Variables with skewed distributions were log-transformed when appropriate. In the models, other covariates (age, sex, time on dialysis, Kt/V, and BMI), were held at their mean values or in proportional distributions, and mean marginal effects were derived. Results are presented as estimated marginal means with 95% confidence intervals, illustrated in figures. When multiple comparisons were performed, *p*-values were adjusted using the Tukey Honest Significant Difference (HSD) method. Statistical analyses for cell experiments were conducted using one–way ANOVA followed by Tukey post hoc test or a paired *t*-test in GraphPad Prism 8.0 (GraphPad Software, USA). Statistical significance was defined as *p* < 0.05. Analyses were conducted in R (v.4.1.1) using the lme4 (v.1.1-28) and ‘emmeans’ packages (v.1.6.2-1) with their dependencies.

## 3. Results

**Figure 1:**
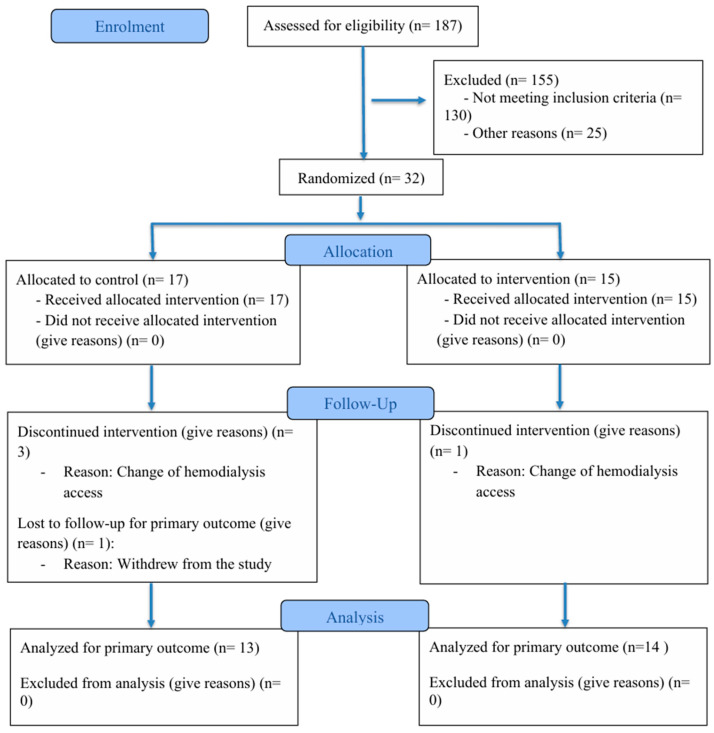
Fluxogram CONSORT.

The Consort flowchart is presented in Figure 1. Baseline demographic and clinical characteristics are presented in Table 1. No significant differences were observed between the groups at either baseline or after the intervention. According to the BMI values, patients were classified as eutrophic according to the WHO classification.

**Figure 2:**
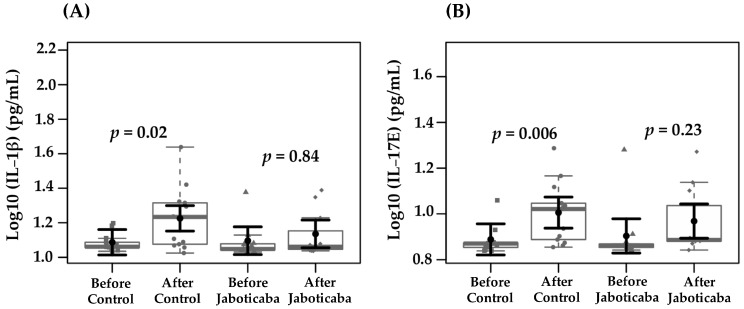
Effects of jaboticaba peel supplementation on serum levels of IL-1β (**A**) and IL-17E (**B**) in patients undergoing hemodialysis. Data sampling distributions are depicted using box-and-strip plots (in grey). The central circle (black) indicates each group expected mean marginal effect, derived from linear mixed-effects models. These models included the intervention group, time, and their interaction as fixed effects, while the patient served as the random effect. Confounding variables included sex, age, hemodialysis duration, Kt/V, and BMI. The black horizontal bars represent the 95% confidence intervals for the expected mean marginal effects per group. The Tukey Honest Significant Difference (HSD) method adjusted *p*-values for multiple comparisons.

Table 2 shows the biochemical parameters assessed at baseline and at the end of the study in both groups. No statistically significant differences were observed either within or between groups. Given that CKD is characterized by chronic low-grade inflammation and oxidative stress, subsequent analyses evaluated whether jaboticaba supplementation could modulate these responses. Table 3 presents the estimated marginal means for oxidative stress, inflammatory, and uremic toxins markers in the control and jaboticaba peel supplementation groups, before and after the intervention. IL-1β levels increased significantly in the control group after the intervention (from 1.08 to 1.22; Δ = +0.14; *p* = 0.02, +13.0%), whereas no significant change was observed in the jaboticaba group (from 1.09 to 1.13 ± 0.04; Δ = +0.04; *p* = 0.84, 3.0%). Also, IL-17 levels increased significantly in the control group after the intervention (from 0.88 to 1.0, Δ = +0.12; *p* = 0.006; +13.3%), whereas a non-significant increase was observed in the jaboticaba group (from 0.9 to 0.96, Δ = +0.07; *p* = 0.23; +7.2%). These findings are illustrated in Figure 2, which shows the distribution of IL-1β and IL-17E levels before and after the intervention.

**Figure 3:**
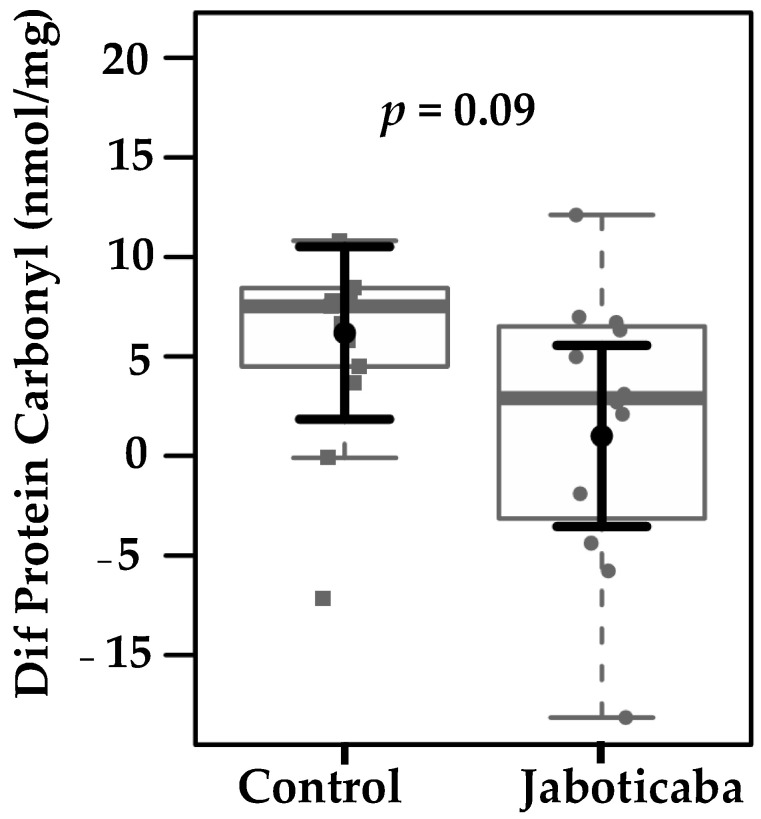
Differences in plasma protein carbonyl levels (nmol/mg) between the control and jaboticaba groups after three weeks of intervention. A trend toward reduced protein carbonylation was observed in the jaboticaba group compared to the Control group (*p* = 0.09). Data sampling distributions are depicted using box-and-strip plots (in grey). The central circle (black) indicates each group expected mean marginal effect—the difference after the intervention—derived from linear fixed-effects models. These models included the intervention group and confounding variables, such as sex, age, hemodialysis duration, Kt/V, and BMI, in their systematic component. The black horizontal bars represent the 95% confidence intervals for the expected mean marginal effects per group.

Since inflammation is closely associated with oxidative damage, the potential of jaboticaba supplementation to reduce protein and lipid oxidation was next evaluated. Plasma protein carbonyl levels in the control and jaboticaba groups were analyzed based on within-group changes (Figure 3). Carbonylated protein levels decreased in the jaboticaba group compared with the control group. The mean change was −3.99 ± 2.16 in the jaboticaba group versus +1.18 ± 2.06 in the placebo group, corresponding to a mean difference of −5.18 ± 2.95 (*p* = 0.09). Although not statistically significant, this result suggests a potential reduction in protein oxidation with jaboticaba supplementation.

**Figure 4:**
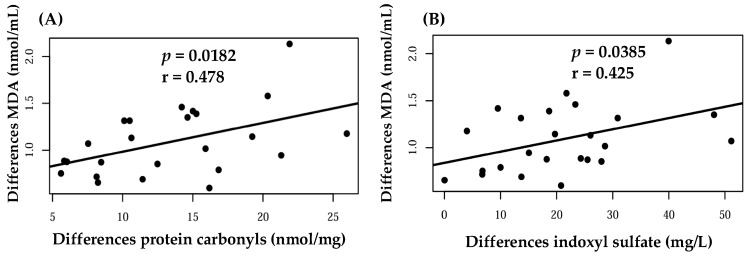
Correlations between differences in plasma malondialdehyde (MDA) levels and (**A**) protein carbonyls and (**B**) indoxyl sulfate. Each dot represents an individual value, and the regression line indicates the association between the variables.

Figure 4 illustrates positive correlations between MDA and protein carbonyl levels (r = 0.478, *p* = 0.0182), as well as between MDA and the uremic toxin IS (r = 0.425, *p* = 0.0385).

To gain further insight into the molecular mechanisms potentially underlying these clinical findings, we reanalyzed publicly available RNA-seq datasets evaluating the effects of ellagic acid, given its high concentration in the peel. The analysis revealed that ellagic acid treatment was associated with the downregulation of several key components of the TLR–MYD88–NF-κB–IL-1 axis. Specifically, differential gene expression analysis demonstrated a significant reduction in the expression of TLR2, TLR4, MYD88, IRAK1, TRAF6, NFKB1, NFKB2, RELA, and IL1B. Furthermore, the expression of downstream effector cytokines, including IL-6 and TNF, was also significantly reduced following exposure to ellagic acid. Conversely, inhibitory regulators such as NFKBIA and NFKBIE showed increased expression levels in the ellagic acid-treated samples (Supplementary Figures S1 and S2).

**Figure 5:**
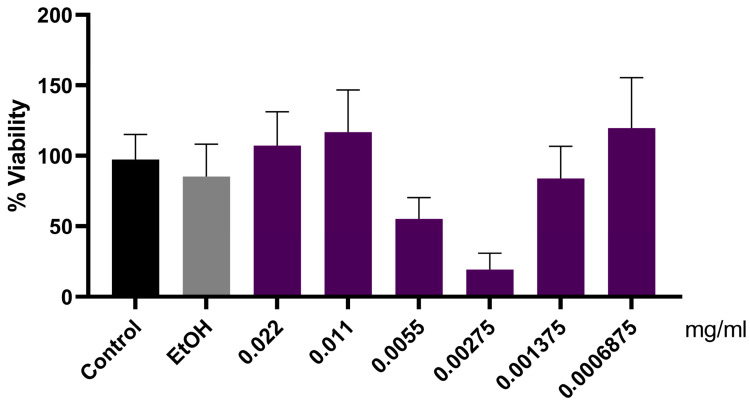
Cell viability assay (MTT) in LLC-PK1 cells. Cells were plated as described in Methods, and jaboticaba peel formulations were added at different concentrations (0.022, 0.011, 0.0055, 0.00275, 0.001375, and 0.0006875 mg/mL). The MMT assay was performed according to the manufacturer’s instructions. Four independent experiments (*n* = 5) were conducted in quintuplicate. Results are expressed as mean ± SEM.

To further clarify whether the observed systemic effects could be directly related to renal cell responses, complementary in vitro assays were performed using porcine kidney proximal tubule cells (LLC-PK1). As shown in Figure 5, the jaboticaba peel formulation suggested a biphasic effect; however, even at high concentrations, no loss of cellular viability was observed.

**Figure 6:**
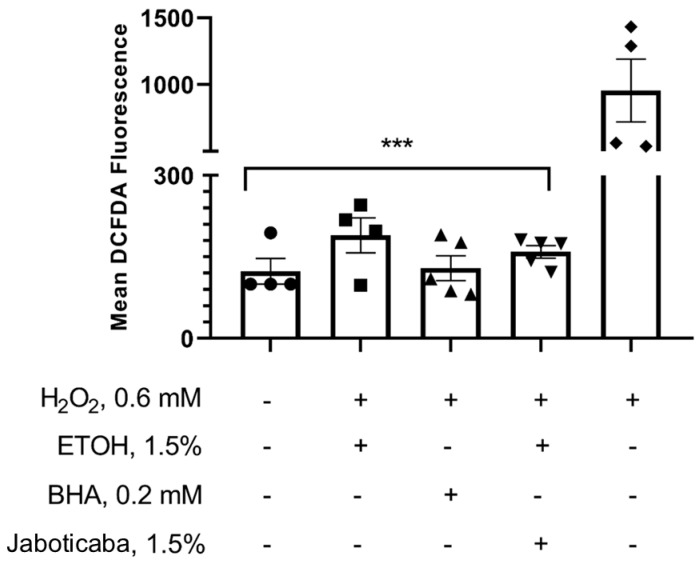
Antioxidant effect of jaboticaba peel formulation in LLC-PK1 cells. Cells were plated as described in the methods, and jaboticaba peel formulation was added to the culture medium at a concentration of 0.0006875 mg/mL. Data are presented as mean ± SEM from four independent experiments, each performed in quintuplicate. Statistical analysis was performed using one-way ANOVA followed by the Tukey post hoc test. *** *p* < 0.0001 *versus* positive control (H_2_O_2_).

To investigate whether the systemic effects of jaboticaba peel are, at least in part, mediated by its antioxidant properties, we evaluated the formulation’s ability to modulate ROS production in renal proximal tubule cells. None of the tested samples increased ROS production. Statistical analysis showed no significant differences among the negative control, ethanol control, antioxidant control (BHA), and jaboticaba. However, when these samples were compared to the positive control (H_2_O_2_), a statistically significant difference was observed. Figure 6 illustrates the relationship between the controls and the jaboticaba peel formulation, consistent with previous findings on the jaboticaba peel antioxidant capacity. Treatment with hydrogen peroxide (H_2_O_2_, 0.6 mM) significantly increased DCFDA fluorescence, indicating enhanced ROS production. The positive control (BHA, 0.2 mM) and jaboticaba peel formulation (1.5%) both reduced fluorescence compared to H_2_O_2_ alone, suggesting antioxidant effects.

## 4. Discussion

This study evaluated the potential of jaboticaba peel supplementation to modulate systemic inflammation and oxidative stress in patients with CKD undergoing HD. The principal findings were that IL-1β and IL-17E levels increased significantly in the control group during the intervention period, whereas no significant changes were observed in the supplemented group. Additionally, a tendency toward reduced protein carbonyl levels was observed following jaboticaba supplementation. These observations collectively suggest that jaboticaba peel may exert protective anti-inflammatory effects in a clinical context characterized by chronic immune activation and heightened oxidative stress. Additionally, cells treated with jaboticaba formulation (1.5%) showed reduced ROS levels, indicating antioxidant activity.

The stabilization of IL-1β and IL-17E levels in the supplemented group is consistent with extensive preclinical evidence demonstrating that jaboticaba bioactive compounds reduce inflammatory signaling. Previous experimental studies have shown that jaboticaba and its by-products can attenuate systemic inflammation. For instance, supplementation with jaboticaba reduced the expression of pro-inflammatory cytokines and key mediators of the NF-κB pathway in animal models [10]. Jaboticaba (*Plinia jaboticaba*) fruit supplementation attenuated colonic inflammation in high-fat-sugar-fed mice by downregulating TNF-α, TLR-4, and NF-κB expression and partially inhibiting inflammasome activation [8]. Similarly, in a rabbit model of atherosclerosis, supplementation with jaboticaba (*P. cauliflora*) peel decreased plasma levels of IL-1β, IL-6, and soluble intercellular adhesion molecule-1 (sICAM-1) and soluble vascular cell adhesion molecule-1 (sVCAM-1) and reduced atherosclerotic lesions [27].

To date, most evidence of its biological effects has come from in vitro and animal studies, which consistently demonstrate antioxidant, anti-inflammatory, and vasoprotective activities. However, studies investigating jaboticaba supplementation in patients with CKD or other clinical conditions remain scarce. Recently, a few clinical trials have begun to explore its potential benefits in human populations, providing preliminary support for its antioxidant and anti-inflammatory effects.

In adults with metabolic syndrome, daily supplementation with 15 g of jaboticaba peel powder for 5 weeks resulted in a significant reduction in postprandial glucose area under the curve, and it decreased circulating IL-6 levels [46]. In healthy individuals, consumption of jaboticaba berry juice (~1300 mg total polyphenols for 6 days) protected against oxidative stress and vascular dysfunction induced by eccentric exercise, preventing declines in flow-mediated dilation, microvascular reactivity, and blood glutathione concentrations [47]. Moreover, a subsequent randomized trial demonstrated that the same jaboticaba juice accelerated muscle recovery and increased plasma GSH levels following exercise-induced muscle damage [48]. Altogether, these clinical findings support the notion that jaboticaba exerts beneficial effects on redox balance, vascular function, and inflammatory biomarkers across different human populations.

The bioactive compounds in jaboticaba peel, particularly anthocyanins (e.g., cyanidin-3-glucoside), ellagic acid, and gallic acid, are known regulators of NF-κB and NLRP3 inflammasome activation. Cyanidin-3-glucoside inhibits IκB-α phosphorylation and degradation, preventing NF-κB nuclear translocation and reducing the downstream transcription of IL-1β, IL-6, IL-8, IL-12, and TNF-α [49]. Ellagic acid also exhibits renoprotective and anti-inflammatory effects. In 5/6 nephrectomized rats, its administration attenuated inflammation, fibrosis, and oxidative stress. These benefits were partly linked to the downregulation of miR-182 and the upregulation of FOXO3a, a transcription factor that modulates genes involved in inflammation and oxidative stress [50]. In a renal ischemia/reperfusion injury, ellagic acid pretreatment attenuated renal dysfunction and histological damage, reduced the levels of TNF-α, IL-1β, IL-6, and monocyte chemoattractant protein-1 (MCP-1), and decreased apoptosis and oxidative stress. These effects were associated with suppression of the NOX4/JAK/STAT signaling pathway [51]. Additional studies have also shown that ellagic acid can inhibit NLRP3 inflammasome activation by downregulating inflammasome-related proteins and limiting ROS production. Under inflammatory stimuli, the NLRP3 inflammasome activates caspase-1, which cleaves pro-IL-1β into its active form, IL-1β [28,52,53,54]. Other proposed mechanisms include the activation of sirtuin 1 (SIRT1) [55] and modulation of nuclear factor erythroid 2-related factor 2 (NRF2)/NF-κB signaling [28], further supporting its pleiotropic renoprotective actions.

Gallic acid, another abundant phenolic compound in jaboticaba peel, exhibits anti-inflammatory and antioxidant properties through multiple pathways. Its effects are primarily due to the inhibition of the NF-κB pathway, possibly through the suppression of phosphorylated IκB-α and NF-κB (p-p65), thereby reducing NF-κB nuclear translocation and the transcription of pro-inflammatory cytokines [56,57]. Gallic acid has also been shown to enhance the AKT/AMPK/Nrf2 pathway, further contributing to redox balance and inflammatory control [56]. In LPS-primed macrophages, gallic acid suppressed mitochondrial ROS (mtROS) production and NLRP3 inflammasome activation [58].

Beyond their anti-inflammatory actions, jaboticaba phenolics enhance antioxidant defenses by reducing ROS production and activating NRF2, leading to the upregulation of antioxidant enzymes, including heme oxygenase-1 (HO-1), nicotinamide adenine dinucleotide phosphate (NAD(P)H) quinone oxidoreductase 1 (NQO1), and glutathione-related enzymes. These effects have been attributed to compounds such as cyanidin-3-glucoside and ellagic acid [59,60,61,62,63]. Flavonols, particularly quercetin and myricetin derivatives, also contribute significantly to jaboticaba’s antioxidant properties. Quercetin can donate electrons from its hydroxyl groups to neutralize ROS, such as superoxide and hydroxyl radicals [64]. Additionally, it inhibits lipid peroxidation, promotes endogenous antioxidant defense systems, and acts as a metal chelator for iron and copper ions, thereby preventing oxidative damage induced by the Fenton reaction [65,66]. This antioxidant response not only mitigates oxidative stress but also prevents the activation of downstream NF-κB and NLRP3 inflammasomes [17,67].

IL-1β plays a central role in CKD-related inflammation, being predominantly secreted by activated monocytes and macrophages, and contributes to renal injury, fibrosis, and cardiovascular complications [68,69]. Elevated IL-1β levels in HD patients have been independently associated with poor clinical outcomes [70]. Thus, the absence of IL-1β elevation in the jaboticaba group may reflect suppression of NF-κB and inflammasome activity by its polyphenols.

IL-17E, or IL-25, is a cytokine member of the IL-17 family that plays a pleiotropic role as a mediator of type 2 immune responses. It induces the production of IL-4, IL-5, IL-13, and thymic stromal lymphopoietin (TSLP) and is released as an alarmin by epithelial and immune cells in response to tissue injury [71]. While some studies suggest a renoprotective role for IL-17E in kidney injury models, others report pro-inflammatory roles depending on the pathological context [72,73,74,75,76,77]. IL-17E signals through IL-17RA/IL-17RB, activating NF-κB, MAPK, JAK, and STAT3 pathways, contributing to inflammation [76,78]. In patients with coronary artery disease, IL-17E expression was increased in both coronary arteries and serum, with higher levels correlating with the severity of coronary stenosis and the occurrence of acute coronary syndrome [75]. The observed stabilization of IL-17E levels in the supplemented group provides additional evidence that jaboticaba phenolics exert anti-inflammatory modulation.

To further elucidate the molecular mechanisms underlying the effects observed in the clinical study, a secondary analysis of public RNA-seq datasets (GSE171311 and GSE171618) was performed, focusing on the transcriptional response to ellagic acid, a primary phenolic compound present in jaboticaba peel, in non-renal cell models [79,80]. Using a curated panel of inflammation-related genes encompassing the TLR, NF-κB, and IL-1 modules, ellagic acid treatment resulted in a marked suppression of key genes within the TLR–MYD88–NF-κB–IL-1 signaling axis, including TLR2, TLR4, MYD88, IRAK1, TRAF6, NFKB1, NFKB2, RELA, and IL1B. Downstream pro-inflammatory cytokines such as IL6 and TNF were also reduced, whereas inhibitory regulators (NFKBIA, NFKBIE) were upregulated, indicating a shift toward an anti-inflammatory transcriptional profile (Supplementary Figures S1 and S2). This transcriptional signature mirrors the stabilization of circulating IL-1β observed in HD patients receiving jaboticaba peel supplementation, suggesting that ellagic acid may exert its effects by attenuating NF-κB-dependent cytokine signaling and modulating NLRP3 inflammasome activation.

Moreover, the observed effects may not only come from the parent compounds but also from metabolites produced by gut microbiota. Anthocyanin- and ellagitannin-derived urolithins exhibit superior bioavailability and potent anti-inflammatory and renoprotective activities, acting by modulating the NF-κB and Nrf2 pathways [4,81,82]. Accordingly, these microbial metabolites may augment the anti-inflammatory profile of jaboticaba polyphenols and partly underlie the inter-individual variability in response to supplementation.

Altogether, these findings provide orthogonal mechanistic support for the clinical outcomes, reinforcing that phenolic compounds from jaboticaba peel, particularly ellagic acid, can downregulate pro-inflammatory signaling at the transcriptional level, potentially contributing to the attenuation of systemic inflammation in CKD.

Regarding oxidative stress, ROS can react with proteins and lipids, leading to the formation of carbonyl groups, aldehydes, and ketones [83]. In this study, protein carbonyls and MDA were measured as markers of oxidative stress. Protein carbonyl levels tended to decrease in the jaboticaba group compared with the control group (*p* = 0.09). MDA correlated positively with both IS and protein carbonyls, reflecting the oxidative stress status in this population. These markers are also associated with increased risk of cardiovascular events [84,85,86]. Previous experimental studies have shown that jaboticaba peel supplementation reduces lipid peroxidation, assessed by MDA, and protein carbonylation [87,88]. The lack of a significant reduction in protein carbonyls and the absence of an effect on MDA in the present study may be attributed to the relatively short intervention period and the administered dosage.

To further investigate whether the antioxidant potential of jaboticaba peel could be directly associated with renal cell responses, complementary in vitro assays were performed using proximal tubule cells (LLC-PK1). The jaboticaba peel formulation did not compromise cell viability, even at higher concentrations, suggesting a favorable safety profile. The biphasic trend observed may be related to concentration-dependent cellular responses to polyphenolic compounds, a phenomenon previously reported for other bioactive compounds [89,90]. Furthermore, the formulation reduced ROS generation induced by H_2_O_2_, demonstrating an antioxidant effect comparable to that of the reference compound BHA, without altering basal ROS levels. These results suggest that the jaboticaba peel formulation has the potential to directly protect renal tubular cells against oxidative stress.

Although systemic oxidative stress markers, MDA and protein carbonyls, were not significantly altered, these in vitro findings suggest that jaboticaba may exert antioxidant effects both through direct actions on renal cells and via systemic modulation. Taken together, these results support the role of jaboticaba as a functional food with renoprotective properties.

The findings of this study are particularly relevant given the high inflammatory and oxidative burden observed in patients with CKD undergoing HD, where chronic activation of inflammatory pathways and oxidative stress contribute to disease progression, cardiovascular events, and increased mortality. In the control group, increased levels of IL-1β and IL-17E may reflect an exacerbated inflammatory state and potentially higher cardiovascular risk. In contrast, the jaboticaba peel-supplemented group showed stable cytokine levels and a trend toward reduced protein carbonyls, suggesting a modulatory effect on both inflammatory and oxidative processes. Although MDA levels did not decrease significantly, the positive correlations observed between MDA and protein carbonyls indicate that these markers accurately reflect oxidative stress in HD patients. These results, together with previous experimental evidence showing that jaboticaba peel can reduce lipid peroxidation and protein carbonylation [87,88], support the potential of this natural intervention as a complementary strategy to mitigate both inflammation and oxidative stress in this high-risk population.

Some limitations of this study should be recognized. The relatively small sample size was partly due to intercurrent events, such as vascular access complications, and participant withdrawal resulting from changes in clinic routines. Moreover, the short intervention period may have limited the extent of the anti-inflammatory effects achieved with jaboticaba peel supplementation. A more extended supplementation period could exert more substantial modulatory effects on the inflammatory profile in patients undergoing HD. Also, the chemical composition of the jaboticaba peel extract was not determined in the present protocol, and the discussion of potential mechanisms was therefore based on evidence from previous analytical studies performed with similar hydroalcoholic extracts. Although these works have consistently identified anthocyanins and other phenolic compounds as the main bioactive constituents, variations in fruit origin, harvest season, and extraction parameters may influence the final phytochemical profile. Moreover, advanced approaches such as network pharmacology or omics-based analyses could further clarify the molecular pathways underlying the observed clinical effects. Future studies combining chemical characterization with systems-level analyses are warranted to strengthen the mechanistic understanding of jaboticaba bioactivity in humans.

Despite these limitations, this is the first clinical study to investigate the impact of jaboticaba peel on inflammation and oxidative stress in this population, providing translational support for previous preclinical findings. Future stages of this research project will involve assessing oxidative stress markers and gene expression to elucidate further the mechanisms underlying the observed effects.

In conclusion, jaboticaba peel supplementation may help mitigate systemic inflammation in patients undergoing hemodialysis, possibly by inhibiting the NF-κB and inflammasome pathways and modulating oxidative stress. These effects were supported by in vitro evidence of cytoprotection and reduced ROS generation in renal cells, as well as transcriptomic signatures consistent with downregulation of pro-inflammatory genes. Taken together, these mechanisms may help stabilize critical cytokines, including IL-1β and IL-17E, both of which are involved in the progression of CKD and in heightened cardiovascular risk. Future investigations with larger cohorts and prolonged intervention periods are required to substantiate these results and better define their clinical implications.

## Figures and Tables

**Table 1:** Baseline demographic and clinical characteristics of patients.

Variables	All (*n* = 27)	Control (*n* = 13)	Jaboticaba (*n* = 14)	*p*-Value *
Age (years)	55 (19.5)	56 (15)	51 (20)	0.64
Sex% (Male/Female)	16/11	8/5	8/6	1.00
Time on HD (months)	26 (48.5)	22 (27)	30.5 (73.2)	0.75
BMI (kg/m^2^)	24.3 (3.7)	24.3 (4.0)	24.3 (3.3)	0.98
Kt/V	1.17 (0.45)	1.14 (0.34)	1.17 (0.72)	0.36

* *p*-values were estimated using non-parametric Mann–Whitney U tests (continuous numerical variables) or Chi-square tests (nominal/categorical variables). Data are presented as median (interquartile range, IQR). Abbreviations: HD: hemodialysis; BMI: body mass index; Kt/V: dialysis adequacy.

**Table 2:** Adjusted marginal means of control and jaboticaba peel supplementation groups before and after intervention in biochemical parameters of CKD patients on HD.

Parameters	Control			Jaboticaba		
	Before	After	*p*-Value *	Before	After	*p*-Value *
Albumin (g/dL)	4.11 (3.98–4.23)	4.12 (3.99–4.25)	0.99	4.08 (3.96–4.20)	4.19 (4.07–4.32)	0.33
Phosphorus (mg/dL)	4.36 (3.51–5.22)	4.55 (3.66–5.44)	0.97	4.85 (4.03–5.66)	4.70 (3.85–5.54)	0.98
Potassium (mmol/L)	5.58 (4.98–6.17)	5.10 (4.48–5.72)	0.37	5.65 (5.12–6.19)	5.18 (4.60–5.75)	0.28
Magnesium (mg/dL)	2.57 (2.32–2.81)	2.43 (2.18–2.67)	0.22	2.45 (2.22–2.68)	2.36 (2.13–2.60)	0.57
Glucose (mg/dL)	93.75 (76.34–111.16)	95.29 (76.78–113.80)	0.99	97.75 (81.52–113.98)	120.06 (103.37–136.75)	0.15

* *p*-values were estimated from contrasts constructed based on average marginal values comparing pre- and post-intervention within each group (control or jaboticaba). Multiple linear mixed-effects models were used to assess time-by-intervention interactions, with patients considered as a random effect. These models included the intervention group and confounding variables, such as sex, age, hemodialysis duration, Kt/V, and BMI, in their systematic component. Estimated marginal means (emmeans) and their 95% confidence intervals were calculated, and the Tukey Honest Significant Difference (HSD) method was applied to adjust *p*-values for multiple comparisons.

**Table 3:** Adjusted estimated marginal means of inflammatory and oxidative stress markers, and uremic toxin levels in CKD patients on HD before and after the intervention period in the control and jaboticaba peel supplementation groups.

Parameters	Control			Jaboticaba		
	Before	After	*p*-Value *	Before	After	*p*-Value *
C-reactive protein (mg/dL)	3.13 (1.6–4.6)	4.16 (2.4–5.8)	0.72	3.59 (2.2–4.9)	3.29 (1.8–4.7)	0.98
Interleukin-1β (pg/mL)	1.08 (1.0–1.1)	1.22 (1.1–1.2)	**0.02**	1.09 (1.0–1.1)	1.13 (1.0–1.2)	0.84
Interleukin-17E (pg/mL)	0.88 (0.8–0.9)	1.00 (0.9–1.0)	**0.006**	0.90 (0.8–0.9)	0.96 (0.89–1.04)	0.23
Malondialdehyde (nmol/mL)	0.95 (0.7–1.1)	1.09 (0,8–1,3)	0.81	0.96 (0.7–1.1)	0.99 (0.7–1.2)	0.99
Protein carbonyl (nmol/mg)	9.54 (6.4–12.6)	10.7 (7.7–13.8)	0.91	14.0 (10.9–17.2)	10.74 (7.5–13.9)	0.31
Indoxyl sulfate (mg/L)	28.23 (18.2–38.2)	24.4 (14.5–34.4)	0.20	22.4 (13.0–31.8)	24.48 (15.0–33.9)	0.63
p-cresyl sulfate (mg/L)	12.45 (3.3–21.5)	10.7 (1.6–19.8)	0.94	21.1 (11.1–31.6)	14.2 (3.9–24.4)	0.14
Indole-3-acetic acid (ug/L)	897.4 (418.1–1376.7)	944.1 (464.8–1423.4)	0.91	1389.1 (888.3–1889.8)	1383.8 (883.1–1884.6)	0.99

* *p*-values were estimated from contrasts constructed based on average marginal values comparing pre- and post-intervention within each group (control or jaboticaba). Multiple linear mixed-effects models were used to assess time-by-intervention interactions, with patients considered as a random effect. These models included the intervention group and confounding variables, e.g., sex, age, hemodialysis duration, Kt/V, and BMI in their systematic component. Estimated marginal means (emmeans) and their 95% confidence intervals were calculated, and the Tukey Honest Significant Difference (HSD) method was applied to adjust *p*-values for multiple comparisons. Bold values indicate statistically significant differences (*p* < 0.05).

## Data Availability

The original contributions presented in this study are included in the article/Supplementary Materials. Further inquiries can be directed to the corresponding author.

## Supplementary Materials

The following supporting information can be downloaded at: https://www.mdpi.com/article/10.3390/foods14234030/s1, Figure S1: Ellagic acid modulates inflammatory gene expression in MDA-MB-231 cells (GSE171618); Figure S2: Ellagic Acid Transcriptomic Effects in HepG2 Cells (GSE171311).

## Abbreviations

The following abbreviations are used in this manuscript:

CKD	Chronic kidney disease
HD	Hemodialysis
ROS	Reactive oxygen species
IAA	Indole-3-acetic acid
p-CS	p-Cresyl sulfate
IS	Indoxyl sulfate
IL	Interleukin
IL-1β	Interleukin-1β
IL-6	Interleukin-6
IL-10	Interleukin-10
IL-17E	Interleukin-17E
TLR-4	Toll-like receptor 4
TNF-α	Tumor necrosis factor-alpha
NF-κB	Nuclear factor-κB
NLRs	NOD-like receptors
NLRP3	NOD-like receptor family pyrin domain containing 3
AVF	Arteriovenous fistula
BMI	Body mass index
FDA	Food and Drug Administration
MDA	Malondialdehyde
WHO	World Health Organization
CRP	C-reactive protein
TBARS	Thiobarbituric acid-reactive substances
TCA	Trichloroacetic acid
DNPH	Dinitrophenylhydrazine
ELISA	Enzyme-linked immunosorbent assay
TMB	Tetramethylbenzidine
HPLC	High-performance liquid chromatography
ATCC	American type culture collection
DMEM	Dulbecco modified eagle medium
FBS	Fetal bovine serum
DMSO	Dimethyl sulfoxide
H2DCFDA	2′,7′-Dichlorofluorescein diacetate
sICAM-1	Soluble intercellular adhesion molecule-1
sVCAM-1	Soluble vascular cell adhesion molecule-1
MCP-1	Monocyte chemoattractant protein-1
SIRT-1	Sirtuin 1
mtROS	Mitochondrial ROS
NRF2	Nuclear factor erythroid 2-related factor 2
HO-1	Heme oxygenase-1
NAD(P)H	Nicotinamide adenine dinucleotide phosphate
NQO1	NAD(P)H quinone oxidoreductase 1
TSLP	Thymic stromal lymphopoietin
