# Supplementary material for: Effects of the Brazilian Native Fruit Jaboticaba (Plinia cauliflora) Peel on Inflammatory and Oxidative Stress Pathways: Insights from a Pilot Study in Hemodialysis Patients and Renal Cell Models
Source: Foods. 2025 Nov 24;14(23):4030. doi: 10.3390/foods14234030 (PMC12692076; doi:10.3390/foods14234030)
Supplement: Supplementary file 1 [file foods-14-04030-s001.zip › foods-3983879-supplementary.pdf]

(A)

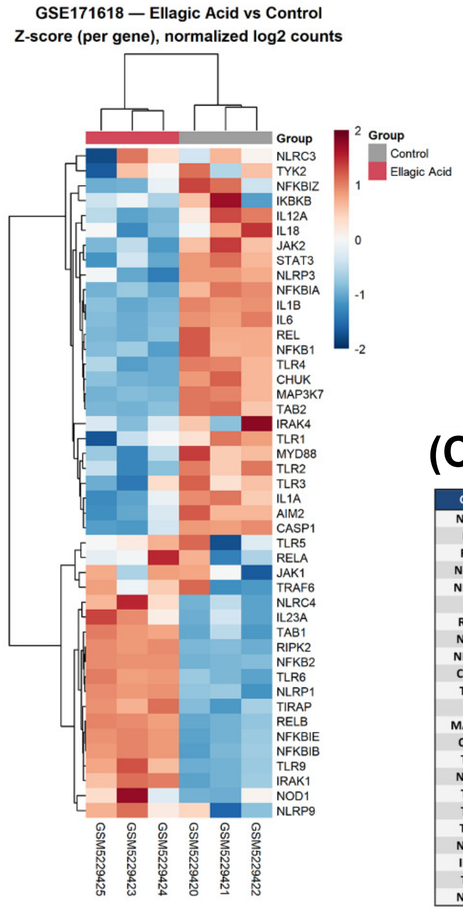

(B)

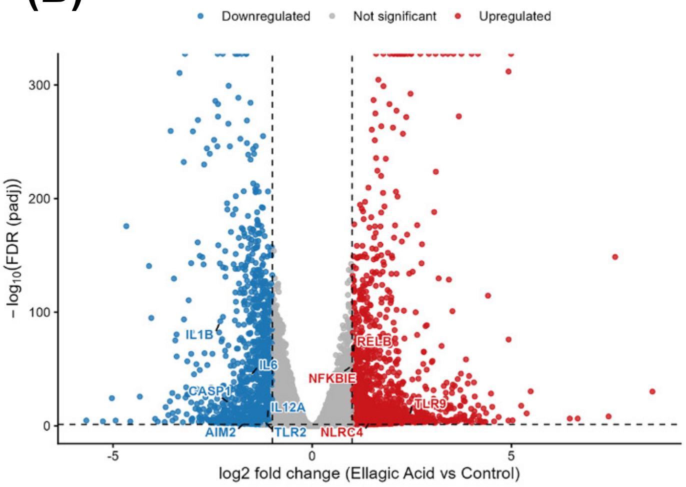

(C)

Gene	Log2FC	pvalue	padj	Regulation
NFKB2	0.94	3.16E-108	1.44E-106	*ns*
IL1B	-2.31	2.54E-94	9.09E-93	Down
RELB	1.03	5.37E-65	1.12E-63	Up
NFKBIE	1.03	1.81E-55	2.98E-54	Up
NFKBIB	0.93	2.98E-51	4.40E-50	*ns*
IL6	-1.57	2.90E-45	3.61E-44	Down
RIPK2	0.73	7.14E-44	8.59E-43	*ns*
NLRP1	0.88	1.64E-35	1.50E-34	*ns*
NFKBIA	-0.75	2.20E-34	1.95E-33	*ns*
CASP1	-2.04	1.18E-20	6.26E-20	Down
TAB2	-0.47	9.33E-20	4.77E-19	*ns*
REL	-0.85	6.24E-18	2.95E-17	*ns*
MAP3K7	-0.35	1.30E-11	4.41E-11	*ns*
CHUK	-0.34	3.02E-11	1.01E-10	*ns*
TLR4	-0.44	5.08E-10	1.57E-09	*ns*
NFKB1	-0.33	1.27E-09	3.85E-09	*ns*
TLR6	0.75	1.84E-09	5.52E-09	*ns*
TLR9	2.45	2.05E-09	6.11E-09	Up
TAB1	0.35	4.38E-07	1.10E-06	*ns*
NLRP3	-0.55	1.73E-06	4.12E-06	*ns*
IL12A	-1.12	2.97E-06	6.96E-06	Down
TLR2	-1.23	5.86E-06	1.34E-05	Down
NLRC4	1.46	1.37E-05	3.02E-05	Up

Gene	Log2FC	pvalue	padj	Regulation
MYD88	-0.30	1.47E-05	3.22E-05	*ns*
JAK2	-0.56	3.37E-05	7.15E-05	*ns*
TIRAP	0.45	8.44E-05	1.72E-04	*ns*
AIM2	-1.68	1.24E-04	2.49E-04	Down
STAT3	-0.16	1.60E-04	3.18E-04	*ns*
TLR3	-0.55	2.75E-03	4.75E-03	*ns*
IL1A	-0.71	2.81E-03	4.84E-03	*ns*
IL18	-0.18	8.46E-03	1.36E-02	*ns*
IL23A	0.97	9.23E-03	1.48E-02	*ns*
IRAK1	0.10	1.06E-02	1.68E-02	*ns*
NFKBIZ	-0.10	7.92E-02	1.09E-01	*ns*
NOD1	0.22	8.45E-02	1.15E-01	*ns*
TLR1	-0.67	1.08E-01	1.44E-01	*ns*
NLRP9	0.86	1.26E-01	1.66E-01	*ns*
IRAK4	-0.09	2.78E-01	3.36E-01	*ns*
RELA	0.06	2.82E-01	3.41E-01	*ns*
TRAF6	0.08	3.18E-01	3.79E-01	*ns*
JAK1	0.06	3.40E-01	4.01E-01	*ns*
IKBKB	-0.04	4.81E-01	5.41E-01	*ns*
TYK2	-0.03	5.55E-01	6.12E-01	*ns*
TLR5	0.12	7.15E-01	7.59E-01	*ns*
NLRC3	-0.01	9.88E-01	9.90E-01	*ns*

Figure S1. Ellagic acid modulates inflammatory gene expression in MDA-MB-231 cells (GSE171618). (A) Heatmap of a curated inflammation gene panel (cytokines, NF-κB axis, TLR/NLR family, JAK/STAT) showing z-score normalized $\log_2$ counts per gene, clustered with Ward.D2 linkage (blue = lower, red = higher expression relative to mean). Columns represent individual biological replicates; top annotation indicates treatment group. (B) Volcano plot of differentially expressed genes in MDA-MB-231 cells treated with ellagic acid (24 h, n=3) versus control (n=3), using DESeq2 (hg38 genome build). Significance thresholds were set at $padj < 0.05$ and $|\log_2 FC| \geq 1.0$. Genes upregulated with ellagic acid are shown in red (right), downregulated in blue (left), and non-significant in grey; significant genes are labeled. (C) Table summarizing DESeq2 differential expression statistics for inflammation genes, including $\log_2$ fold change, standard error, test statistic, p-value, and FDR ($padj$).

(A)

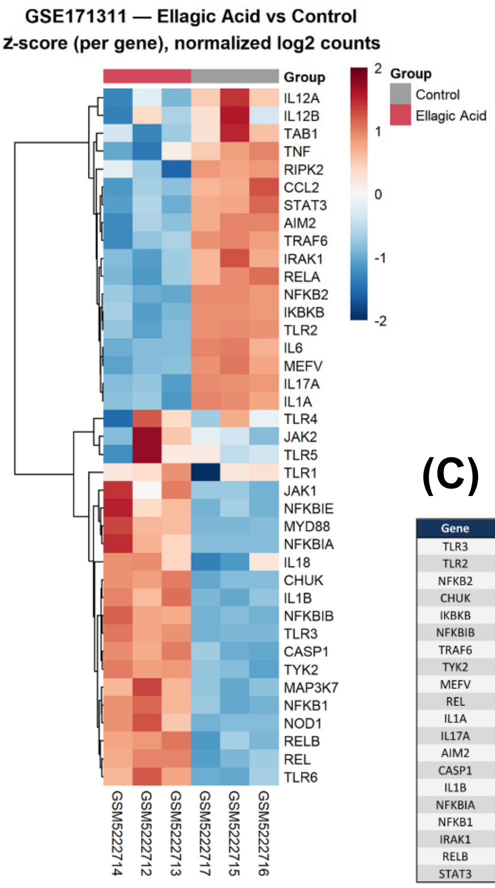

(B)

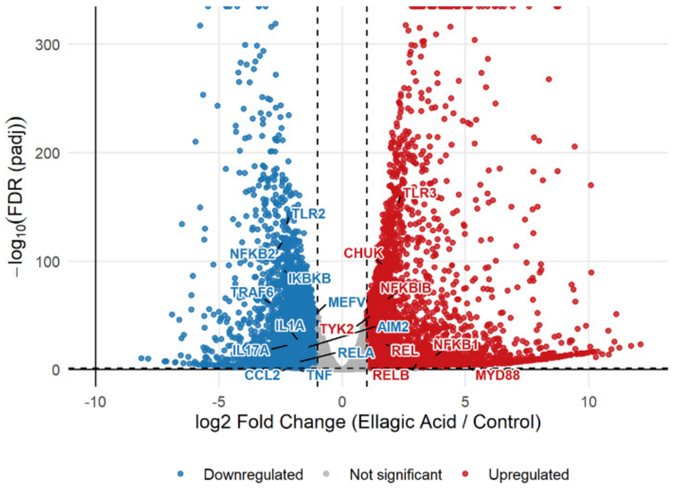

(C)

Gene	log2FC	pvalue	padj	Regulation
TLR3	2.25	5.82E-153	2.80E-151	Up
TLR2	-2.27	9.90E-134	3.77E-132	Down
NFKB2	-2.37	3.21E-122	1.06E-120	Down
CHUK	1.75	2.32E-97	5.22E-96	Up
IKBKB	-2.42	2.06E-96	4.58E-95	Down
NFKBIB	1.70	1.05E-64	1.30E-63	Up
TRAF6	-2.78	9.01E-61	1.02E-59	Down
TYK2	1.26	1.75E-53	1.72E-52	Up
MEFV	-1.12	1.89E-52	1.80E-51	Down
REL	1.60	5.70E-27	2.73E-26	Up
IL1A	-1.70	1.45E-26	6.89E-26	Down
IL17A	-2.03	3.60E-25	1.63E-24	Down
AIM2	-1.57	4.69E-21	1.83E-20	Down
CASP1	0.72	7.96E-20	2.96E-19	*ns*
IL1B	0.81	1.81E-15	5.64E-15	*ns*
NFKBIA	0.87	8.89E-14	2.56E-13	*ns*
NFKB1	3.66	6.49E-12	1.71E-11	Up
IRAK1	-0.45	2.21E-10	5.43E-10	*ns*
RELB	3.08	2.29E-09	5.29E-09	Up
STAT3	-0.59	4.01E-08	8.68E-08	*ns*

Gene	log2FC	pvalue	padj	Regulation
RELA	-1.92	3.55E-07	7.23E-07	Down
MYD88	5.01	5.51E-07	1.10E-06	Up
IL6	-0.84	1.28E-05	2.31E-05	*ns*
RIPK2	-0.77	1.54E-05	2.76E-05	*ns*
TLR6	0.74	1.39E-04	2.30E-04	*ns*
NFKBIE	0.37	2.89E-04	4.66E-04	*ns*
JAK1	0.27	4.28E-04	6.80E-04	*ns*
NOD1	0.77	4.57E-04	7.23E-04	*ns*
CCL2	-2.12	1.26E-03	1.91E-03	Down
IL12A	-0.25	1.01E-02	1.40E-02	*ns*
TNF	-1.87	1.27E-02	1.74E-02	Down
MAP3K7	0.26	1.53E-02	2.08E-02	*ns*
IL18	0.13	8.05E-02	9.96E-02	*ns*
TAB1	-0.13	9.79E-02	1.20E-01	*ns*
JAK2	0.47	1.47E-01	1.76E-01	*ns*
IL8	-0.11	2.51E-01	2.88E-01	*ns*
TLR5	0.21	4.97E-01	5.38E-01	*ns*
IL12B	-0.27	6.31E-01	6.68E-01	*ns*
TLR1	0.16	6.53E-01	6.87E-01	*ns*
TLR4	0.03	9.30E-01	9.38E-01	*ns*

Figure S2: Ellagic Acid Transcriptomic Effects in HepG2 Cells (GSE171311). (A) Heatmap of expression for a curated panel of inflammation-related genes in ellagic acid–treated vs. control HepG2 samples. Each row is a gene, and each column is a sample (three biological replicates per condition). Gene expression values (DESeq2-normalized counts) are $\log_2$-transformed and then Z-score normalized per gene (row). Blue, below-average gene expression, and red, above-average gene expression. (B) Volcano plot of differential expression results from DESeq2, plotting each gene's $\log_2$fold change (EA vs Control) on the x-axis versus the $-\log_{10}$(adjusted p-value) on the y-axis. Genes meeting the significance criteria (padj < 0.05 and $|\log_2$fold change$| \geq 1$) are shown as filled points (colored), while non-significant genes are in gray. Members of the inflammation gene panel are highlighted and labeled by gene name on the plot. (**C**) Summary table of the DESeq2 differential expression statistics for the selected inflammation-related genes. For each gene, the $\log_2$fold change (EA treatment vs control) and the adjusted p-value (padj) are reported.
